# Mechanisms of pathological synchrony in Parkinson’s disease induced by changes in synaptic and cellular properties due to dopamine

**DOI:** 10.1186/1471-2202-13-S1-P54

**Published:** 2012-07-16

**Authors:** Choongseok Park, Leonid L Rubchinsky

**Affiliations:** 1 Department of Mathematical Sciences and Center for Mathematical Biosciences, Indiana University Purdue University Indianapolis, Indianapolis, IN 46202, USA; 2 Present address: Department of Mathematics, University of Pittsburgh, Pittsburgh, PA15260, USA; 3 Stark Neurosciences Research Institute, Indiana University School of Medicine, Indianapolis, IN 46202, USA

## 

Synchronized oscillations between neurons or across nuclei within parkinsonian basal ganglia (BG) have been extensively reported over past decade. They are implied in the hypokinetic motor symptoms of Parkinson’s disease [1]. Thus, it is crucial to understand the mechanisms underlying the observed abnormally synchronous rhythms. Previous experimental studies have indicated that two nuclei within BG, subthalamic nucleus (STN) and external globus Pallidus (GPe), may comprise key substrates in the maintenance of the pathological synchronous rhythms in parkinsonian BG. Modeling studies also demonstrated that STN-GPe network has an ability to generate various rhythms due to the reciprocal inhibitory-excitatory connections [2]. Although other structures may also play important roles, recent modeling work [3] suggests that STN-GPe network are able to exhibit characteristic intermittent temporal patterns of synchrony observed in experimental recordings from parkinsonian patients [4].

Dopamine loss, a characteristic feature of Parkinson’s disease generally accepted to cause motor symptoms, is known to affect network properties as well cellular properties of STN-GPe network. In this study, we considered how these changes due to dopamine loss contribute to the generation of pathological rhythms in STN-GPe network. We reduced large STN-GPe network model [2] (each nuclei consist of several model neurons described by experimentally-based conductance-based equations) to two simplified models. First one is a network consisting of two inhibitory bursting cells with self-inhibition and the second one is GPe neuron with the periodic STN synaptic input. The dynamics of and bifurcations in the models were studied by computational and analytical techniques.

There is an experimental evidence for how dopamine presynaptically suppresses different types of basal ganglia synapses. We show how the chance in strength of dopamine-modulated synapses promotes intermittent synchronous activity patterns under very general conditions and investigate the cellular properties, necessary for this dynamics. It is also known that dopamine affects not only synaptic properties, but also cellular properties of BG neurons. In particular, change in the dopaminergic status leads to change in the temporal features of firing patterns of STN neuron. So we also considered how dopamine-dependent features of STN neuron firing modulate the GPe response and the synchrony between STN input and GPe output. As STN dopaminergic modulation and STN activity pattern vary, we observe the overall transition from low coherence state to high coherence state through intermittent synchronous activity. This points to the important role of GPe cells within BG circuit and demonstrate that the response of GPe cell, which was achieved through the interaction between the intrinsic properties of the cell itself and membrane properties of STN cell, provide good source for the temporal characteristics of synchrony observed in experimental setting.

In sum, our modeling studies explain how the lack of dopamine in Parkinson’s disease may lead to the generation of intermittent synchronous dynamics in the basal ganglia via two different pathways: cellular and synaptic.

## References

[B1] Hammond C Bergman H Brown P Pathological synchronization in Parkinson's disease: networks, models and treatments Trends Neurosci 2007 30 357 364 10.1016/j.tins.2007.05.004 17532060

[B2] Terman D Rubin JE Yew AC Wilson CJ Activity patterns in a model for the subthalamopallidal network of the basal ganglia J Neurosci 2002 22 2963 2976 11923461 10.1523/JNEUROSCI.22-07-02963.2002 PMC6758326

[B3] Park C Worth RM Rubchinsky LL Neural dynamics in parkinsonian brain: the boundary between synchronized and nonsynchronized dynamics Phys Rev E 2011 83 042901 10.1103/PhysRevE.83.042901 PMC3100589 21599224

[B4] Park C Worth RM Rubchinsky LL Fine temporal structure of beta oscillations synchronization in subthalamic nucleus in Parkinson's disease J Neurophysiol 2010 103 2707 2716 10.1152/jn.00724.2009 20181734 PMC2867579

